# Differential regulation of neutrophil degranulation by p38 MAPK and mitochondrial ROS during *Mycobacterium leprae* infection

**DOI:** 10.3389/fphar.2026.1883257

**Published:** 2026-08-12

**Authors:** Isabella Forasteiro Tavares, Rafaela de Souza Andrade, Natasha Cabral, Roberta Olmo Pinheiro, Veronica Schmitz

**Affiliations:** Fundação Oswaldo Cruz, Fiocruz, Instituto Oswaldo Cruz, Laboratório de Hanseníase, Rio de Janeiro, Brazil

**Keywords:** inflammatory response, leprosy, low density neutrophils, *Mycobacterium leprae*, neutrophilic degranulation

## Abstract

**Introduction:**

Erythema Nodosum Leprosum (ENL) is an inflammatory complication of leprosy that is associated with the progression of peripheral nerve damage and the resultant physical disabilities. Neutrophil-mediated inflammation plays a crucial role in the emergence of ENL. Recent evidence suggests that *Mycobacterium leprae* triggers neutrophil degranulation and the generation of Low-Density Neutrophils (LDN). This study aimed to investigate whether inhibition of critical regulators of human neutrophil activation can regulate *M. leprae*-induced neutrophil degranulation.

**Methods:**

To explore the mechanisms underlying inflammation *in vitro*, whole-blood cultures were incubated with* M. leprae*. The effects of selective inhibitors in *M. leprae* co-cultures were subsequently evaluated by measuring surface expression of granule markers, levels of inflammatory cytokines, and release of granule-related proteins, including MMP-9, PTX3, and MPO.

**Results:**

We found that at least 8 h of *M. leprae* incubation was necessary to trigger primary and secondary granule degranulation, while tertiary granules began to degranulate within 4 h. At this 4-h mark, LDNs were observed in cell cultures. By 24 h, we confirmed neutrophil degranulation and LDN generation. We observed a correlation between the release of pro-inflammatory cytokines and degranulation markers. The secretion of IL-8 and IL-6 was associated with secondary and tertiary granule degranulation. Selective inhibition of p38 MAPK affected the release of tertiary and secondary granules as well as IL-6 secretion. Moreover, mito-TEMPO treatment showed that mitochondrial ROS production seems to be crucial for *M. leprae*-induced primary and secondary granules degranulation. Finally, the inhibition of TGF-β receptor 1 indicates that TGF-β modulates *M. leprae*-induced neutrophil degranulation and LDN generation through binding to the receptor.

**Discussion:**

These results indicate that p38 MAPK pathway and mitochondrial ROS production are important contributors to *M. leprae*-induced neutrophil degranulation, while TGF-β/TGF-βR1 signaling exerts an important regulatory effect. These findings advance our knowledge of the molecular mechanisms underlying *M. leprae*-induced degranulation and raise the possibility of new potential therapeutic strategies for targeting ENL-related degranulation.

## Graphical Abstract

**Figure:**
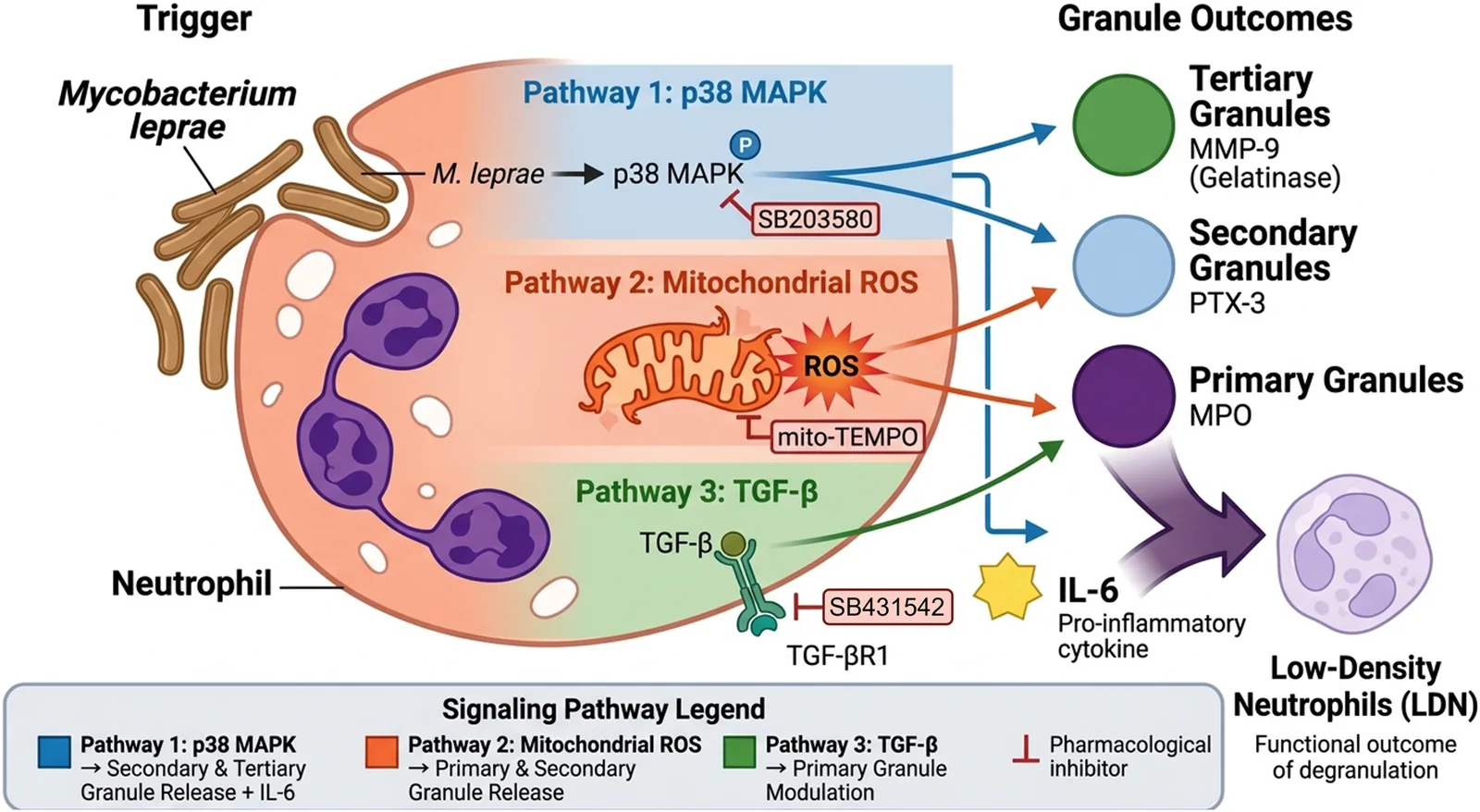
Molecular mechanism. Source: SciSpace AI-generated infographic (July 2026).

## Introduction

Leprosy is a chronic infectious disease caused by the intracellular Mycobacterium *leprae* and *Mycobacterium lepromatosis.* This disease primarily affects the skin and peripheral nerves (Britton and Lockwood, 2004). In 2024, 172,717 new cases of leprosy were reported globally, resulting in a case detection rate of 21.1 per million. This underscores the urgent need to address leprosy as a significant health issue in endemic areas, where new cases continue to be reported annually (World Health Organization, 2025). Rapid diagnosis and early treatment with multidrug therapy (MDT), which effectively eliminates *M. leprae*, are essential for controlling transmission and preventing nerve damage and disabling lesions.

Erythema Nodosum Leprosum (ENL) is an inflammatory complication of leprosy that affects Borderline Leprosy (BL) and Lepromatous Leprosy (LL) (Ridley and Jopling, 1966), comprising the multibacillary (MB) group. ENL may occur before, during, or after MDT (Nery et al., 1998; Voorend and Post, 2013). ENL is presented by a single episode, but frequently some patients experience a chronic condition with recurrent episodes (Walker et al., 2015). It is attributed to the immune stimulation induced by whole or fragmented *M. leprae*. Indeed, bacillary index (BI) > 4+ is considered a risk factor for ENL.

Clinically, ENL is characterized by painful subcutaneous erythematous nodules and is most often accompanied by systemic symptoms such as malaise, fever, and neutrophilic leukocytosis (Polycarpou et al., 2017). The presence of neutrophils in ENL lesions has long been considered an essential factor for diagnosis. But more recent reports have emphasized the role of circulating neutrophils in ENL systemic inflammation, including their spontaneous ability to release tumor necrosis factor (TNF) (Pacheco et al., 2020) and neutrophil extracellular traps (NETs) (Da Silva et al., 2019), as well as an increased neutrophil-to-lymphocyte ratio (NLR) in patients with moderate/severe ENL (Feitosa da Silva Barboza et al., 2024). These findings highlight the significant involvement of neutrophils in the ENL immunological/inflammatory process.

One of the main effector mechanisms of neutrophils is degranulation, which results in the release of inflammatory proteins stored in neutrophilic granules. Neutrophil granules are generally classified as azurophilic (primary), specific (secondary), and gelatinase (tertiary) granules, which contain proteins such as myeloperoxidase (MPO), pentraxin-3 (PTX-3), and matrix metalloproteinase-9 (MMP-9), respectively (Sheshachalam et al., 2014). The release of these proteins contributes to antimicrobial defense but may also amplify inflammation and promote tissue damage when dysregulated.

Recently, we described that patients with ENL exhibit a distinct subset of low-density neutrophils (LDNs) in the mononuclear cell fractions of peripheral blood (Tavares et al., 2021). Although the origin and role of LDNs require further investigation, evidence suggests that these cells may contribute to the pathogenesis of ENL due to their activated profile (CD11b^High^ CD62L^Low^), enhanced degranulation ability, and high frequency in severe ENL cases. We also presented *in vivo* evidence of the ability of *M. leprae* to induce neutrophilic degranulation, such as the presence of an increased number of proteins commonly found in neutrophil granules, like PTX-3 and MMP-9, in the serum of leprosy patients (Mendes et al., 2017; Teles et al., 2010). Additionally, we observed gene activation of degranulation pathways (Leal-Calvo and Moraes, 2020; Rosa et al., 2024) and a correlation between the frequency of circulating LDNs and levels of degranulated MMP-9 in the setting of ENL (Tavares et al., 2021). Altogether, neutrophil degranulation is an essential factor in the immunopathogenesis of ENL. Nonetheless, many gaps remain in our understanding of the interactions between neutrophils and *M. leprae*.

Therefore, the present study is crucial in our ongoing efforts to understand the role of neutrophils and *M. leprae* interactions, investigating the ability of dead *M. leprae* to induce degranulation and the signaling pathways related to this mechanism.

## Materials and methods

### Study participants

Healthy volunteers were recruited at the Leprosy Laboratory, Oswaldo Cruz Foundation, Rio de Janeiro, RJ, Brazil. Eligible participants were informed verbally about the study and invited to participate. Written consent was obtained from all participants before peripheral whole blood heparin samples collection. This study protocol was approved by the Ethics Committee of the Oswaldo Cruz Foundation (CAAE 56113716.5.0000.5248).

### Whole blood cultures

To perform the *in vitro* degranulation and LDN generation assays, 1–2 mL of whole blood from healthy individuals were added per well in microplates. In the first step, whole blood cultures were stimulated with 10 μg/mL of dead *M. leprae*, either sonicated *M. leprae* (MLS; NR-19329, BEI Resources), consisting of a pool of *M. leprae* components, after bacilli disruption by sonication, or irradiated *M. leprae* (MLI; NR-19326, BEI Resources), consisting of gamma-irradiated whole bacilli with preserved cellular structure. Cultures stimulated with dead *M. leprae* were performed for 4–24 h at 37 °C, 5% CO2. Cultures using 1 mL of whole blood were infected with Multiplicity of Infection (MOI) 10:1 of live *M. leprae* (strain 48 Thai-5) purified from the paws of athymic mice, kindly provided by Dr. Patrícia Sammarco from the Lauro de Souza Lima Institute (Bauru, SP). Cultures containing live *M. leprae* were maintained for 24 h at 33 °C, 5% CO2, conditions considered optimal for preserving bacillary viability. For all experiments, unstimulated controls, consisting of whole blood cultured under identical conditions but without *M. leprae,* were included. In the second stage, cultures were pretreated or not with 10 ng/mL of Recombinant Human Transforming Growth Factor-beta 1 (TGFβ1; AF-100-21C, Peprotech), 10 µM of the TGF-β receptor 1 inhibitor, SB431542 (1,614, Tocris) or 10 µM of mitoTEMPO (SML0737, Sigma-Aldrich) for 1 h. Cultures were also pretreated or not with 10 µM of the selective p38 MAPK inhibitor SB203580 (S8307), 20 μg/mL of α1-antitrypsin (A1AT; A6150), or 5 µM of TPEN (P4413) for 30 min (all purchased from Sigma-Aldrich), followed by stimulation with 10 μg/mL of MLS and MLI for 24 h. At the end, all blood samples were processed by Ficoll-Hypaque gradient as previously described by Tavares et al. (2021). Plasma supernatants were collected and stored at −20 °C for degranulation measurements. Cells were analyzed by multiparametric flow cytometry.

### Multiparametric flow cytometry

The frequency of LDNs (1 × 10^6^ to 1.5 × 10^6^ cells) generated during *in vitro* assays were analyzed in the peripheral blood mononuclear cells (PBMC) fraction by flow cytometry. LIVE/DEAD near-IR fluorescent reactive dye (L10119, Invitrogen) was added 30 min before labeling. Cells were stained in the presence of Human FcR Blocking for 30 min at 4 °C and protected from light with or without the following mixture of fluorescent-labeled anti-human antibodies: CD14-FITC (11-0149-42) or PercP-Cy5.5 (45-0149-42) from eBioscience; CD15-Pacific Blue (323024); CD63-PE (353004); CD66b-AlexaFlour700 (305,113) from Biolegend. Cells were washed, fixed in 2% paraformaldehyde, and stored protected from light at 4 °C until the moment of acquisition. The BD FACSymphony A5 cytometer (BD Biosciences) from the Fiocruz Technology Platform was used to acquire samples. Approximately 50,000 events per sample were acquired within the granulocyte region using the SSC x FSC parameters. The data were analyzed using FlowJo software (BD Biosciences). LDNs were characterized within the PBMC gate as CD15^+^ CD14^−^ cells (Tavares et al., 2021). Degranulation was analyzed by measuring the percentage of positive cells and median fluorescence intensity (MFI) of CD63 and CD66b on the cell surface of LDNs. Fluorescence Minus One (FMO) was adopted as a control sample without labeling the receptors of interest.

### ELISA measurement

Degranulation was measured by concentrations of Myeloperoxidase (MPO–DY3174), Pentraxin-3 (PTX-3 – DY 1826) and Matrix-Metalloproteinase-9 (MMP-9 - DY911) in culture supernatants via enzyme-linked immunosorbent assay kits (ELISA), according to the manufacturer’s protocol (all purchased from R&D Systems). This assay detected total MMP-9 in both its active and *pro-forma* form. Levels of the pro-inflammatory cytokines IL-1β (900-T95), IL-8 (900-K18), IL-6 (900-K16) and TNF-α (900-T25) were also evaluated (all purchased from Peprotech). Values represent the mean of duplicate determinations in each sample. The minimum and maximum detection levels of the kits are 31.2–2000 pg/mL for MMP-9; 62.5–4,000 pg/mL for MPO; 219–14,000 pg/mL for PTX-3; 16–1,000 pg/mL for IL-8; 24–1,500 pg/mL for IL-6; 8–1,000 pg/mL for IL-1β and TNF-α. Total degranulation was calculated as the sum of MPO, PTX-3, and MMP-9 concentrations measured in culture supernatants. Absorbance values were measured using the SpectraMax 190 microplate reader (Molecular Devices) and compared with the standard curve to calculate concentration rates using the SoftMax Pro v5.3 program (Molecular Devices).

### Statistical analysis

The analyses were performed using GraphPad PRISM software, version 8 (GraphPad Software). Data are presented as mean ± SEM. Individual donor values are displayed in all graphs. The D'Agostino-Pearson normality test was applied to observe data distribution. To compare the results of the two groups, the unpaired Mann-Whitney test and the Wilcoxon test for paired samples of non-parametric distribution were utilized. To contrast more than two groups of non-parametric samples, the Kruskal–Wallis test followed by Dunn’s multiple comparison test was carried out. Spearman or Pearson correlation coefficients were used when appropriate, depending on data distribution. The differences were considered significant when p < 0.05.

## Results

### *Mycobacterium leprae* induces degranulation in a time-dependent manner

**FIGURE 1:**
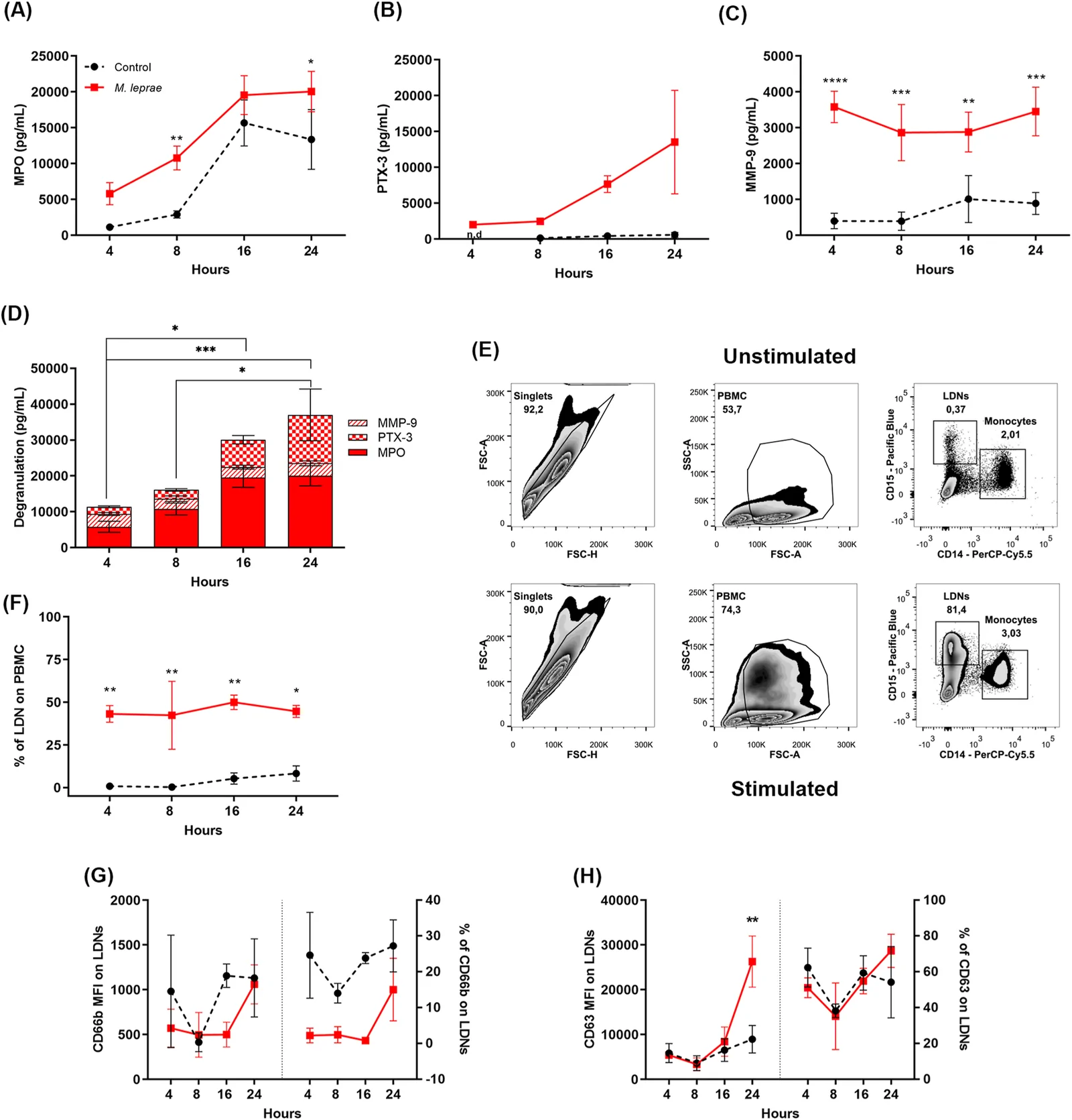
Neutrophilic degranulation kinetics induced by dead *M. leprae in vitro*. Whole blood cells were cultured either in the absence of *M. leprae* (unstimulated control) or stimulated with 10 μg /mL of sonicated *M. leprae* for 4 h, 8 h, 16 h, and 24 h. Granule proteins were measured on plasma culture supernatants by ELISA, and translocation of degranulation markers to the plasma membrane of LDNs was assessed by flow cytometry. Concentration of **(A)** MPO **(B)** PTX-3 **(C)** MMP-9 (pg/mL), at different time-points **(D)** Total degranulation levels (MPO, PTX-3 and MMP-9) in stimulated cultures **(E)** Graphs representative of the flow cytometry gating strategy used to identify LDNs in the PBMC samples of an unstimulated compared to a stimulated sample. The first gate was set on FCS-A and FSC-H to eliminate doublets, then on PBMC based on SSC-A and FCS-A parameters. LDNs were analyzed in the PBMC gate based on the CD15^+^CD14^−^ profile **(F)** Frequency of generated LDNs. Median of fluorescence intensity (MFI) and percentage (%; Lines and dots) of **(G)** CD66b and **(H)** CD63 translocation to LDNs plasma membrane. Combined data from 3 independent donors, each analyzed in triplicate, are shown. Each point and/or bars represents mean ± SEM per group. Statistics: Two-way ANOVA. *p < 0.05, **p < 0.01, ***p < 0.001, ****p < 0.0001.

Knowing that there is evidence of degranulation in leprosy and that 24 h of stimulus with sonicated dead *M. leprae* is capable of inducing generation of LDNs *in vitro* (Tavares et al., 2021), we analyzed MPO, PTX-3, and MMP-9, proteins related to primary, secondary, and tertiary neutrophilic granules, respectively, in plasma supernatant of whole blood cultures stimulated *in vitro* with dead *M. leprae* at different times. MPO levels had a time-dependent increase, but the same was observed in unstimulated cultures (whole blood cultured in the absence of *M. leprae*), suggesting that some MPO are released spontaneously (Figure 1A). PTX-3 was detected in unstimulated samples only after 8 h of culture, and was increased in stimulated cultures also after 8h, reaching a higher peak in the 24-h period (Figure 1B), although no significant differences were observed. In contrast, a significant increase in MMP-9 levels was seen at all time points analyzed (Figure 1C). When we combined the three neutrophilic granule proteins, a significant increase in total degranulation was observed after 16 h of *M. leprae* stimulus. (Figure 1D). Analysis of granule marker expression on the cell surface is another tool for characterizing neutrophil degranulation. Therefore, to investigate if the subpopulation of LDNs observed in the context of *M. leprae* infection is originated by degranulation, we evaluated the frequency of LDNs generated *in vitro* and the translocation of granule markers CD63 and CD66 b to the plasma membrane of LDNs through multiparametric flow cytometry. The frequency of LDNs was analyzed using the CD15^+^CD14^−^ labeling profile (Figure 1E), and the results showed that, compared with unstimulated controls, the presence of *M. leprae* induced the generation of LDNs *in vitro* at all time points evaluated (Figure 1F). *M. leprae* also increased the expression of the primary granule marker CD63 on LDNs’ plasma membrane when compared to unstimulated control after 24 h (Figure 1H), but no differences in the expression of the secondary/tertiary granule marker CD66b were detected (Figure 1G). No statistical difference was observed regarding the percentage of LDNs expressing CD66B or CD63 (Figures 1G,H).

**FIGURE 2:**
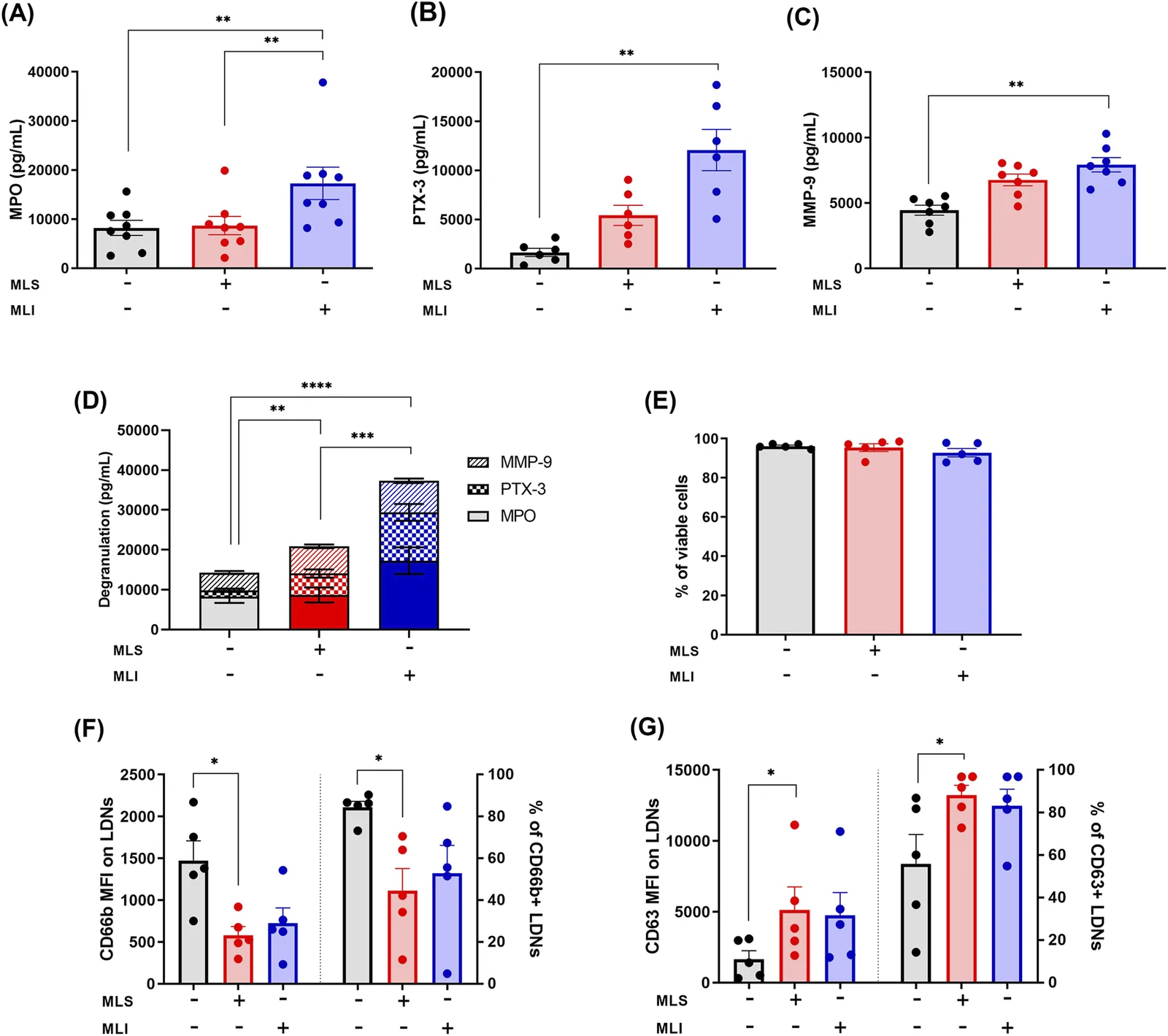
Comparison of the ability of sonicated and irradiated dead *M. leprae* to induce neutrophilic degranulation *in vitro*. Whole blood cells were cultured either in the absence of *M. leprae* (unstimulated control) or stimulated with 10 μg /mL of *M. leprae* sonicated (MLS) or irradiated (MLI) for 24 h. Granule proteins were measured by ELISA on plasma culture supernatants, and translocation of degranulation markers to the plasma membrane of LDNs was assessed by flow cytometry. Concentration of degranulated **(A)** MPO **(B)** PTX-3 **(C)** MMP-9 (pg/mL) **(D)** Total degranulation levels (MPO, PTX-3, and MMP-9) in stimulated cultures **(F)** Frequency of viable LDNs in stimulated cultures determined by live-dead staining. Median of fluorescence intensity (MFI) and percentage (%) of **(F)** CD66b and **(G)** CD63 translocation to LDNs plasma membrane. Combined data from 7 independent experiments. Each point represents an individual donor, and bars represent mean ± SEM per group (n = 5–8). Statistics: One-way ANOVA. *p < 0.05, **p < 0.01, ***p < 0.001.

To investigate whether there is a difference in the degranulation-inducing capacity between *M. leprae* components or by structurally intact dead bacilli, we compared the response after sonicated (MLS) or irradiated (MLI) *M. leprae* 24 h stimulation, the peak of the degranulation process as seen in Figure 1D. MLI increased MPO levels compared to MLS and unstimulated control (Figure 2A). PTX-3 and MMP-9 levels were also increased after MLI stimulation (Figures 2B,C). MLS and MLI increased total degranulation levels compared to unstimulated control (Figure 2D). The frequency of viable LDN cells did not change in the presence of the MLS or MLI (Figure 2E), but the expression of degranulation markers did. CD66b expression was reduced on the surface of LDNs generated by MLS, and the percentage of CD66b^+^ cells compared to the unstimulated control (Figure 2F). In contrast, we observed an inverse profile of CD63, i.e., a significant increase in the percentage of CD63 expressing LDNs, as well as in their expression on LDNs compared to unstimulated control (Figure 2G). No difference among the expressions of the two markers analyzed was found after MLI stimulus (Figures 2F,G).

### Induction of neutrophilic degranulation by live *Mycobacterium leprae*

**FIGURE 3:**
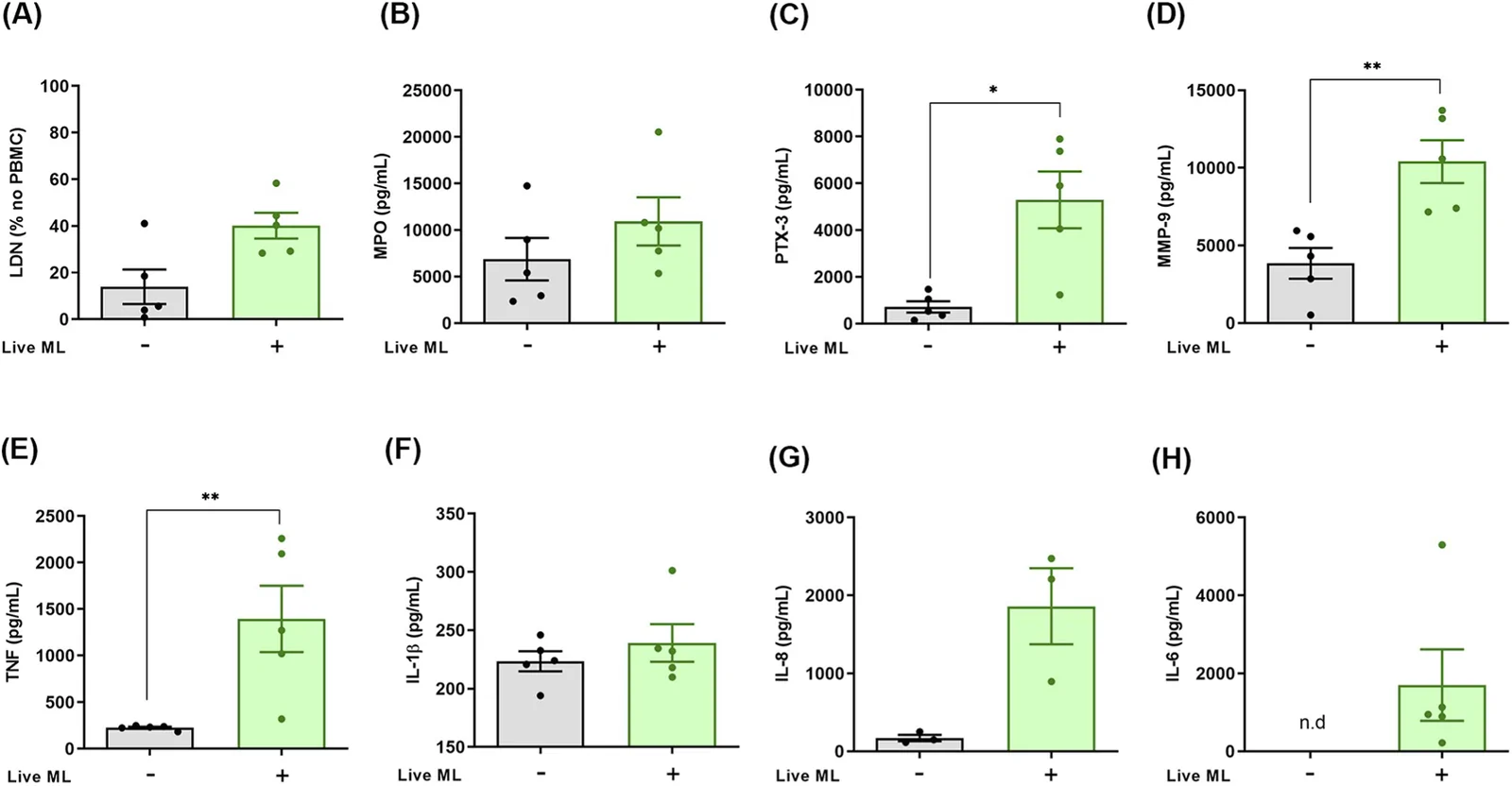
Effect of live *M. leprae* infection on neutrophilic degranulation and pro-inflammatory cytokines release *in vitro*. Whole blood cells were cultured in the absence of *M. leprae* (unstimulated control) or infected with live *M. leprae* (MOI 10:1) for 24 h. Granule proteins and cytokines release were measured by ELISA on plasma culture supernatants, and frequency of LDNs was assessed by flow cytometry **(A)** Frequency of generated LDNs. Concentration of **(B)** MPO **(C)** PTX-3 **(D)** MMP-9 **(E)** TNF **(F)** IL-1 **(G)** IL-8 and **(H)** IL-6 (pg/mL). Combined data from 3 independent experiments. Each point represents an individual donor and bars represent mean ± SEM per group (n = 5). Non-detected: n. d. Statistics: T-test. *p < 0.05, **p < 0.01.

To assess the biological relevance of findings obtained with dead *M. leprae*, we next ed whether live *M. leprae* could also trigger neutrophil degranulation and, consequently, generate LDNs *in vitro*. For this, we incubated live *M. leprae* in whole blood cultures for 24 h and evaluated proteins related to neutrophilic granules as well as the profile of cytokines released in the plasma supernatant. As shown in Figure 3, we noticed that the frequency of LDNs was increased around 3-fold in the presence of live *M. leprae* (mean [standard error]: 40.12 [5.50]) compared to uninfected control (mean [standard error]: 13.97 [7.39]) (Figure 3A), and MPO, PTX3 and MMP9 were significantly elevated in cultures infected with live *M. leprae* (Figures 3B–D). Despite no significant difference in IL-1β, IL-8, and IL-6 release (Figures 3F–H), the presence of live *M. leprae* increased TNF secretion (Figure 3E). Due to the inability to cultivate *M. leprae in vitro*, which restricts access to live bacilli, subsequent experiments were performed using dead *M. leprae* preparations.

### Inhibition of p38 MAPK by SB203580 reduces degranulation of secondary and tertiary granules

**FIGURE 4:**
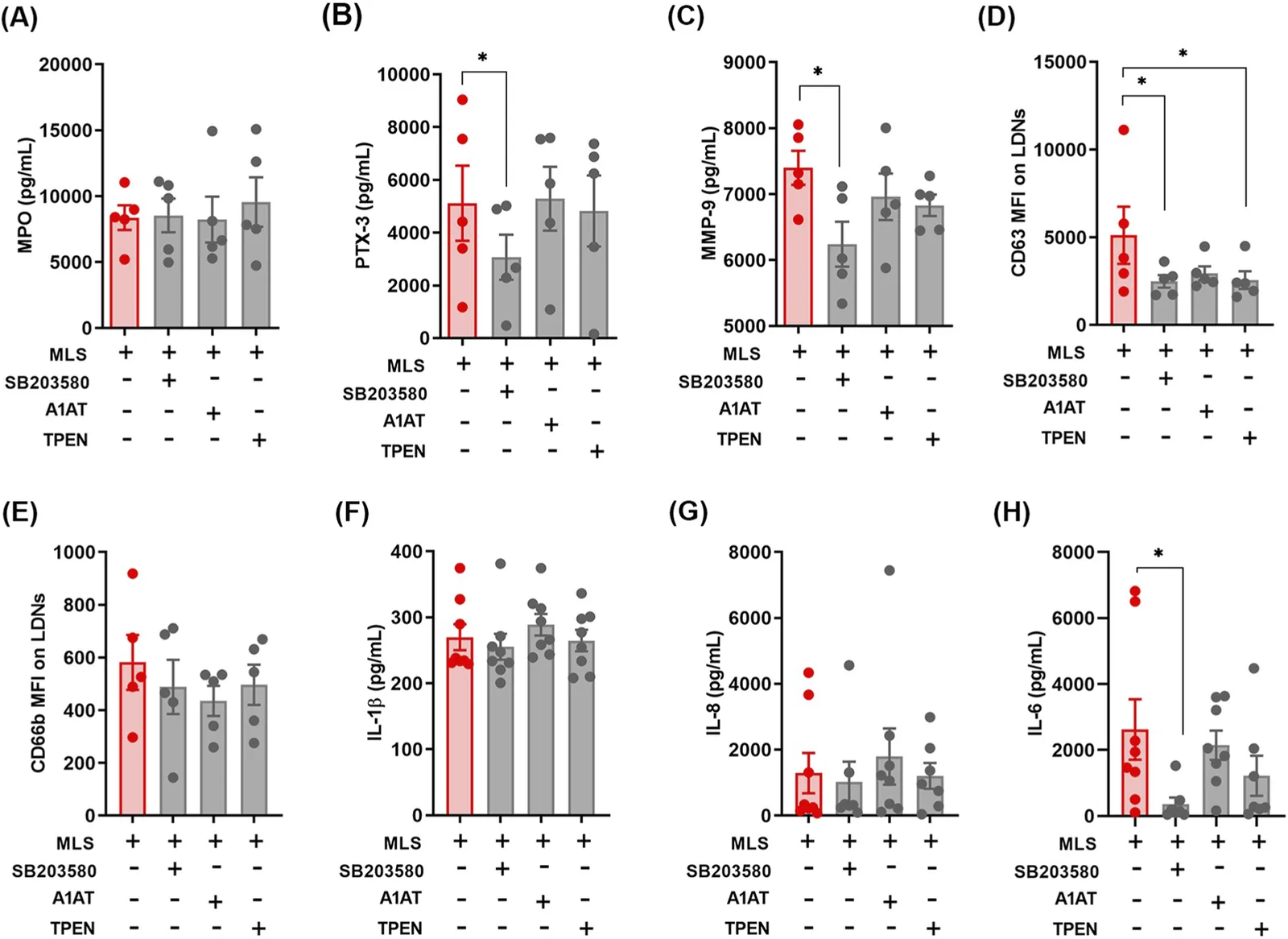
Effect of SB203580, A1AT, and TPEN inhibitors on degranulation and cytokine release mediated by *M. leprae in vitro.* Whole blood cells were cultured with MLS alone or pretreated with SB203580, A1AT or TPEN, before stimulation with MLS for 24 h. Granule proteins and cytokines release were measured by ELISA on plasma culture supernatants, and translocation of degranulation markers to the plasma membrane of LDNs was assessed by flow cytometry. Concentration of **(A)** MPO **(B)** PTX-3 **(C)** MMP-9 **(F)** IL-1β **(G)** IL-8 and **(H)** IL-6 (pg/mL). Median of fluorescence intensity (MFI) of **(D)** CD63 and **(E)** CD66b translocation to LDNs plasma membrane Combined data from 6 independent experiments. Each point represents an individual donor and bars represent mean ± SEM per group (n = 5–8). Statistics: One-way ANOVA. *p < 0.05, **p < 0.01.

Recent studies have highlighted an enhancement in the activation of degranulation pathways in patients with ENL (Leal-Calvo and Moraes, 2020; Rosa et al., 2024). Therefore, to identify molecular pathways that may be involved in the improvement of degranulation induced by MLS, we pretreated whole blood cultures with drugs that act on pathways capable of inhibiting degranulation. We analyzed the effect of SB203580, a selective p38 Mitogen-Activated Protein Kinase (MAPK) inhibitor, on the key serine protease inhibitor Alpha-1-antitrypsin (A1AT) and on the NETs release inhibitor TPEN, a membrane-permeable zinc chelator. All inhibitor-treated conditions were compared with cultures stimulated with MLS alone, which served as the reference condition. Degranulation of the primary granule marker MPO was not altered with the presence of any inhibitor (Figure 4A). Inhibition of p38 MAPK by SB203580, led to a reduction in both secondary and tertiary granule markers degranulation induced by MLS (Figures 4B,C). Translocation of CD63 to the plasma membrane of LDNs decreased in the presence of SB203580 and TPEN (Figure 4D), but no difference was observed regarding CD66b expression (Figure 4E). A similar profile was observed in samples stimulated with MLI (Supplementary Figure S1). Furthermore, thalidomide, the drug of choice for controlling ENL, did not appear to exert a significant effect on primary, secondary, or tertiary granule exocytosis mediated by MLS (Supplementary Figure S2). However, given the limited number of donors analyzed, no definitive conclusions can be drawn regarding the effect of thalidomide on neutrophil degranulation. Since previous studies indicate that cytokine release occurs in parallel with degranulation (Pellmé et al., 2005; Naegelen et al., 2015a), we evaluated the levels of IL-1β, IL-8, and IL-6, implicated in the cytokine storm observed in leprosy reactions. Pretreatment with degranulation inhibitors had no significant effect in the secretion of IL-1β and IL-8 (Figures 4F,G). However, the inhibition of p38 MAPK by SB203580 reduced the secretion of IL-6 induced by MLS (Figure 4H).

### Suppression of mitochondrial ROS production decreases degranulation of primary and secondary neutrophil granules

**FIGURE 5:**
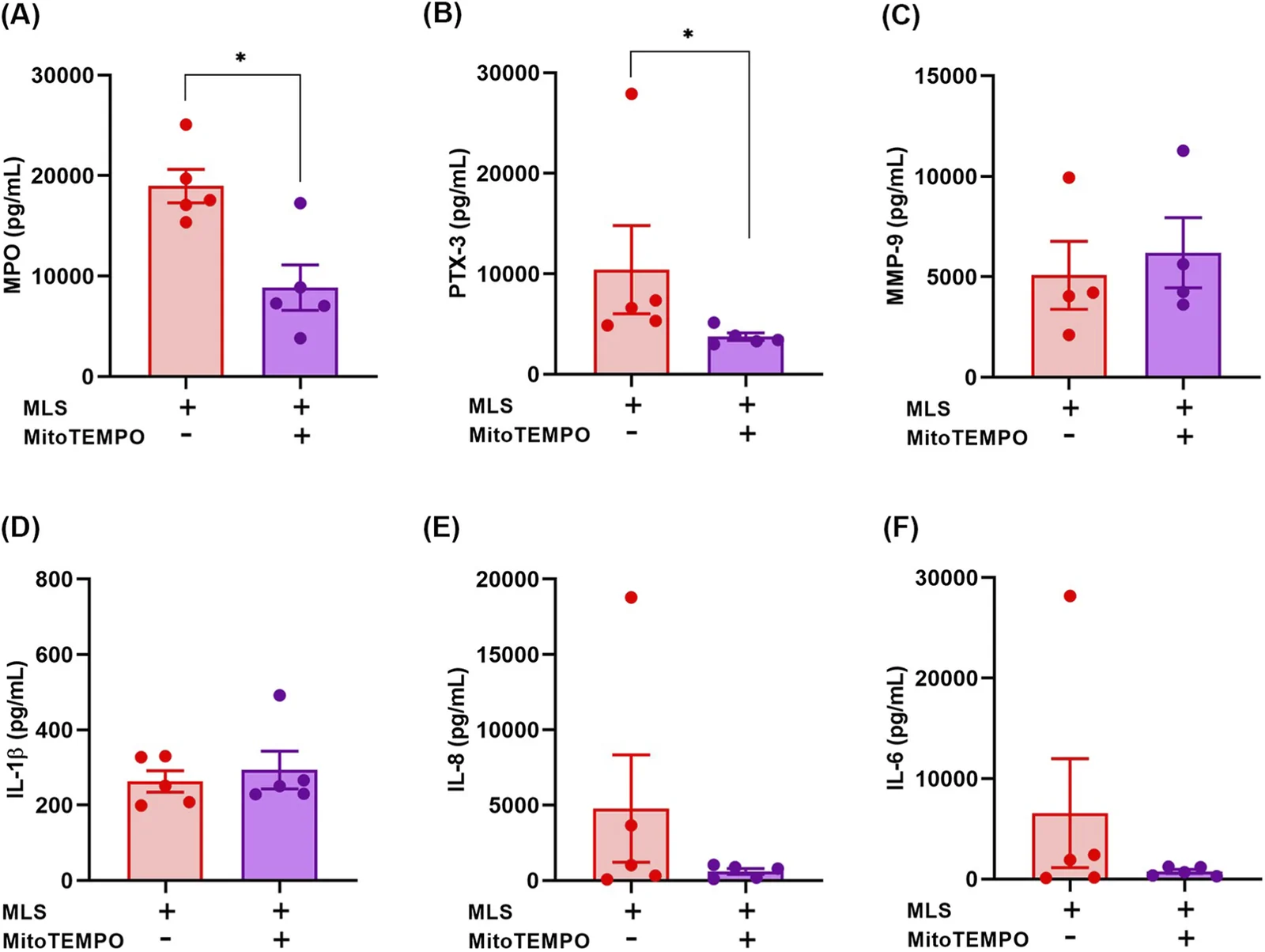
Suppression of mitochondrial ROS production impacts *M. leprae*-induced degranulation *in vitro.* Whole blood cells were cultured with MLS alone or pretreated with mito-TEMPO before stimulation with MLS for 24 h. Granule proteins and cytokines release were measured by ELISA on plasma culture supernatants. Concentration of **(A)** MPO **(B)** PTX-3 **(C)** MMP-9 **(D)** IL-1β **(E)** IL-8 and **(F)** IL-6 (pg/mL). Combined data from 3 independent experiments. Each point represents an individual donor and bars represent mean ± SEM per group (n = 4–5). Statistic: One-way ANOVA. *p < 0.05.

In our previous paper, transmission electron microscopy analysis showed that the presence of mitochondria was more abundant in the cytoplasm of circulating LDNs from leprosy patients (Tavares et al., 2021). Knowing that mitochondrial Reactive Oxygen Species (mROS) may be critical for the degranulation of human neutrophils (Vorobjeva et al., 2017), we investigated whether the suppression of mROS production with Mito-TEMPO, one mitochondria-targeted oxidant, could impact *M. leprae*-induced degranulation. Compared with cultures stimulated with MLS alone, cultures pretreated with Mito-TEMPO prior to MLS stimulation exhibited a marked reduction in the exocytosis of primary (MPO) and secondary (PTX-3) granules (Figures 5A,B). No change in MMP-9 degranulation (Figure 5C), as well as IL-1β release (Figure 5D) was observed. Still, although not significant, the means indicates that Mito-TEMPO reduced by about 8-fold the secretion of IL-8 (mean [standard error]: 603.6 [192.3]) and IL-6 (mean [standard error]: 754.8 [198.7]) compared to *M. leprae-*stimulated cultures (mean [standard error]: IL-8 - 4775 [3560]; IL-6 - 6554 [5425], respectively) (Figures 5E,F).

### TGF-β modulates primary neutrophil granule degranulation via TGF-β receptor 1 signaling

**FIGURE 6:**
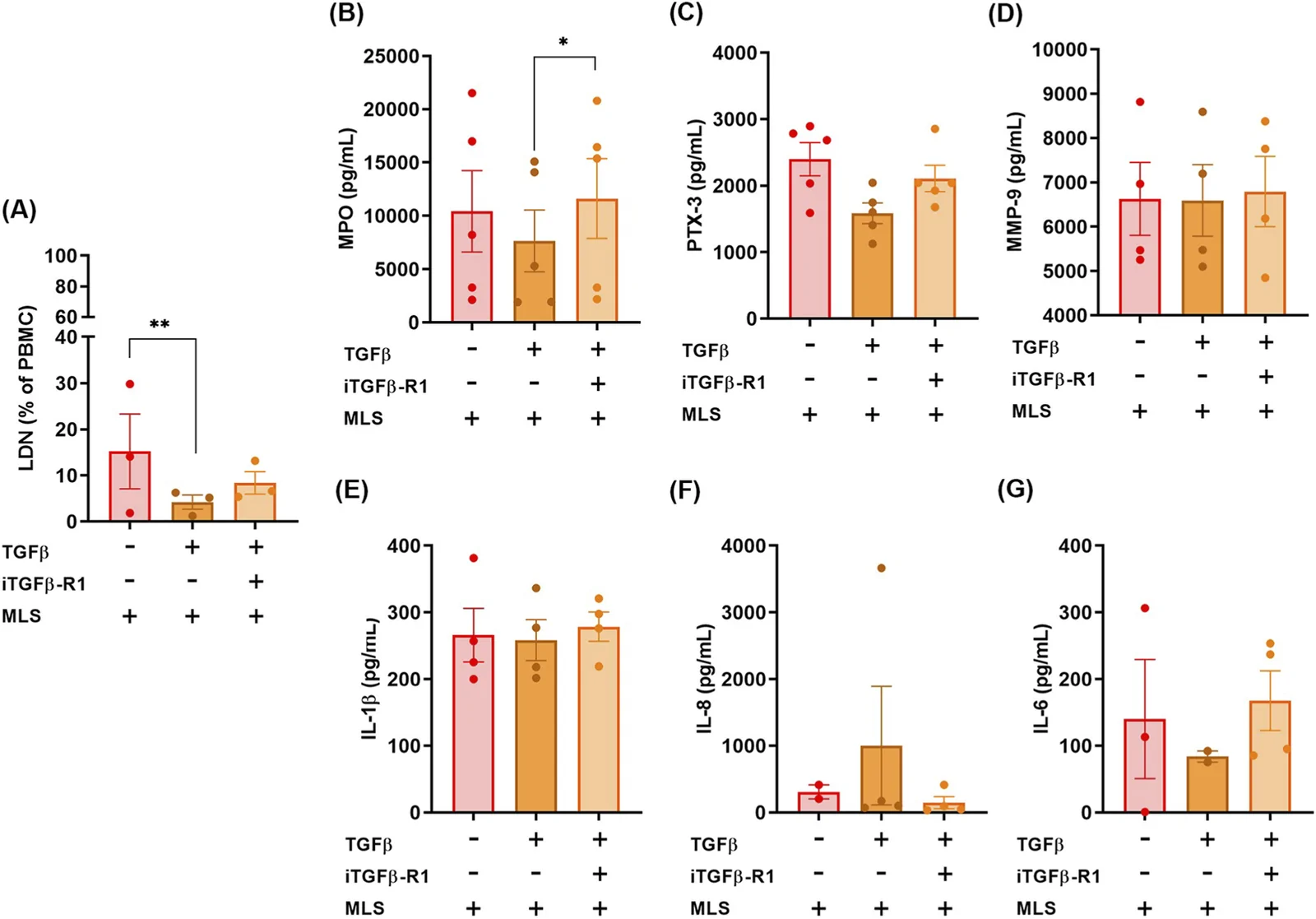
TGF-β inhibits LDN generation and *M. leprae*-mediated primary granule degranulation via TGF-β receptor 1. Whole blood cells were cultured with MLS alone, pretreated with recombinant TGFβ alone or in combination with an inhibitor of TGF-β receptor 1 (iTGFβ1-R1) before stimulation with MLS for 24 h. Granule proteins and cytokines release were measured by ELISA on plasma culture supernatants, and the frequency of LDNs was assessed by flow cytometry **(A)** Frequency of generated LDNs. Combined data from 2 independent experiments, with each data point performed in triplicate (n = 3). Concentration of **(B)** MPO **(C)** PTX-3 **(D)** MMP-9 **(E)** IL-1β **(F)** IL-8 and **(G)** IL-6 (pg/mL). Combined data from 4 independent experiments. Each point represents an individual donor and bars represent mean ± SEM per group (n = 4–5). Statistic: One-way ANOVA. *p < 0.05, **p < 0.01.

The immunosuppressive cytokine TGF-β, described as an inhibitor of neutrophil degranulation (Shen et al., 2007) is known to interfere with the generation of LDNs mediated by MLS (Tavares et al., 2021). To further examine if the effect of TGF-β occurs through modulation of degranulation via TGF-β binding to TGF-β receptor 1, whole blood cultures were stimulated with MLS alone, TGF-β + MLS, or TGF-β + MLS following pretreatment with SB431542, a specific TGF-β receptor 1 inhibitor (iTGF-βR1). The inhibition of TGF-βR1 was able to attenuate the modulatory effect of TGF-β on the generation of LDNs by MLS (Figure 6A). Besides, our results suggest that although the inhibition of TGF-βR1 does not appear to affect MMP-9 degranulation (Figure 6D), it attenuated the degranulation of MPO and PTX-3 modulated by TGF-β (Figures 6B,C), with a significant effect on MPO, restoring their levels toward those observed in cultures stimulated with MLS alone. The release of IL-1β was not affected by any experimental conditions tested (Figure 6E). Although with no significant differences, the results suggest that TGF-β modulates the release of IL-8 (Figure 6F) and IL-6 (Figure 6G), and TGF-βR1 inhibition could reverse this modulation to levels similar to the ones observed in cultures stimulated with MLS alone.

**FIGURE 7:**
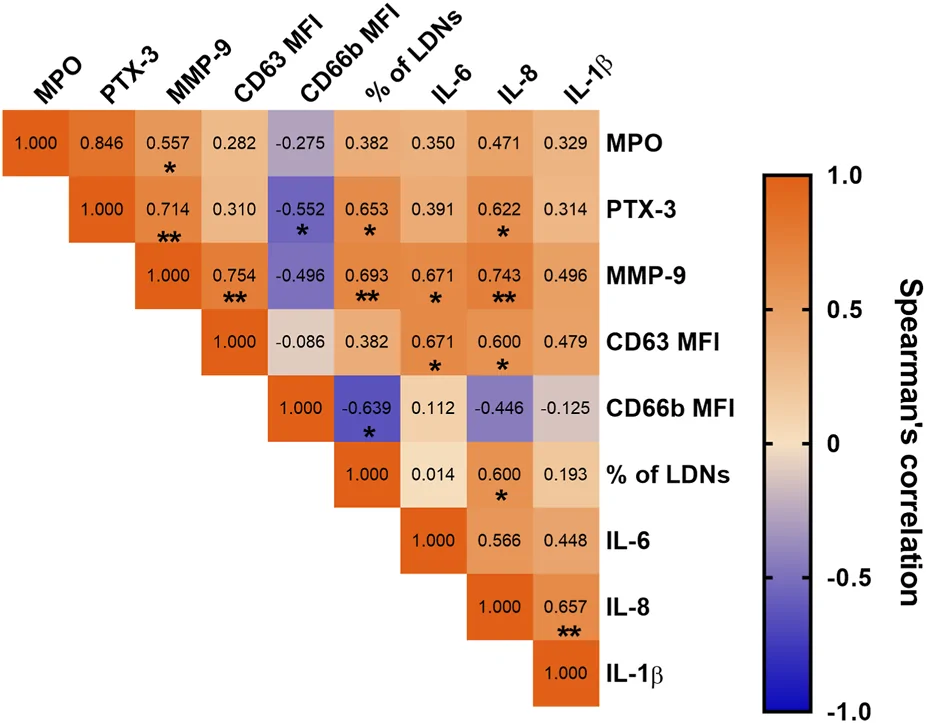
Triangular heatmap of Spearman correlation coefficient analyses. Spearman’s r correlation coefficient ranges between −1.0 and 1.0. The orange color on the heatmap indicates positive correlation, while the blue color indicates negative correlation. The stronger the coloration, the higher the correlation coefficient (n = 15). Correlation coefficients and p‐values are given in the matrix. *p < 0.05, **p < 0.01.

Spearman’s correlation was used for a correlation-heatmap between markers of neutrophil degranulation (MPO, PTX-3, MMP-9, CD63, and CD66 b), frequency of LDNs, and the release of IL-6, IL-8, and IL-1β (Figure 7). The analysis indicates a significant positive association between MMP-9 with MPO, PTX-3, CD63 expression, frequency of LDNs, and the cytokines IL-6 and IL-8. Besides, PTX-3 was also positively associated with the frequency of LDNs and the release of IL-8. The release of the cytokines IL-6 and IL-8 was positively related to the expression of CD63, IL-8, and the frequency of LDNs. Furthermore, the levels of IL-1β and IL-8 were positively correlated. A negative association was observed between CD66b expression, the level of PTX-3, and the frequency of LDNs in the samples.

## Discussion

The results presented in this study offer new insights into the relationship between *M. leprae* and neutrophils, as *M. leprae* (dead irradiated/sonicated, or live) induced degranulation and LDN emergence *in vitro*. Neutrophil granules formed from the trans-Golgi network during neutrophil maturation are divided into primary, secondary and tertiary granules and undergo an ordered inverse release based on stimulus intensity (Sheshachalam et al., 2014). Degranulation induced by *M. leprae* starts with tertiary granules being released early, and primary and secondary granules require a more vigorous or prolonged stimulus. It is well established that tertiary granules are the most sensitive to stimulation, with about 30%–40% of their proteins being released extracellularly *in vitro* and *in vivo* (McLeish et al., 2017; Yin and Heit, 2018). Interestingly, degranulation of secondary granules (PTX-3) occurred only after 16h, suggesting the need for priming, as reported in the literature (McLeish et al., 2017; Potera et al., 2016). Phenotypical analysis revealed an increase in CD63 (primary granule marker) and a reduction in CD66 b (secondary granule and adhesion receptor) in LDNs of MLS cultures. The exacerbation of pro-inflammatory immune responses observed in COVID-19 is associated to excessive neutrophil activation, and the pattern of CD63 and CD66b expression is similar to the one observed in our study (Parackova et al., 2020). CD66 b expression was also reduced on neutrophil cell membrane after fMLP and CXCL8 exposure (Kim and Haynes, 2013). Furthermore, works showed that neutrophils release soluble CD66b (CEACAM8) upon stimulation, whether by proteolytic shedding, receptor internalization, or microvesicle release (Zhao et al., 2004; Suades et al., 2024). Besides, there is evidence that extracellular chromatin, like the one derived from NETosis present in ENL leprosy (Da Silva et al., 2019), can trigger the secretion of soluble CEACAM8 by primary human neutrophils (Ribon et al., 2019). These mechanisms could be related to the CD66 b membrane expression patterns observed in our study.

Investigating the mechanisms related to the induction of neutrophil degranulation by *M. leprae*, we demonstrated that p38 MAPK pathway may be involved in *M. leprae-induced* degranulation, since the SB203580 inhibitor attenuated secondary and tertiary granules degranulation, along with the reduction of the primary granule marker CD63 on LDNs. The inhibition of p38 MAPK has been proven to be efficient in controlling the exocytosis of tertiary granules after TNF stimulation, and of primary and secondary granules after stimulation with fMLP primed with TNF, indicating an essential role of MAPKs in this process (McLeish et al., 2017). ERK inhibition had no significant effect, and treatment with A1AT did not impact primary degranulation, indicating that the HcK pathway may not be involved in this context. Although the effects of SB203580 support the involvement of p38 MAPK in *M. leprae*-induced secondary and tertiary granules release, p38 MAPK activation was not directly assessed in the present study. Thus, the contribution of p38 MAPK pathway was inferred from the effects of pharmacological inhibition.

Previously, signs of degranulation, increased endoplasmic reticulum (ER) and mitochondrial profiles, indicative of cellular stress, were described in LDNs of ENL patients (Tavares et al., 2021). We demonstrate that mitochondrial ROS production is essential for *M. leprae-induced* degranulation, as mito-TEMPO treatment significantly reduced primary and secondary granule release, corroborating the results of Vorobjeva et al. (2017), which described mitochondrial ROS as important for the exocytosis of these granules.

Furthermore, in our work, blocking TGF-β receptor 1 with the ALK5 inhibitor SB431542 reversed the modulatory effects of TGF-β on MPO degranulation and LDN generation *in vitro*, indicating that the TGF-β/TGFβR1 axis mediates TGF-β′s suppressive activities in neutrophils stimulated with *M. leprae.* In ENL, a decrease in TGF-β-producing regulatory T cells (Tregs) (CD4+TGF-β+ and CD8+TGF-β+) was reported (Gomes de Castro et al., 2022), suggesting an environment permissive to exacerbated degranulation due to decreased TGF-β levels, and possibly an improved frequency of LDNs in the *ex vivo* ENL setting. Our data reveal that MMP-9 and PTX-3 supernatant levels are positively correlated with LDN frequency, suggesting a central role of degranulation of secondary and tertiary granules in its generation. Previous studies also reported increased MMP-9 (Teles et al., 2010) and PTX-3 (Mendes et al., 2017) levels in lesions of ENL patients, which may favor the development of ENL by boosting cutaneous inflammation through endothelial activation and extracellular matrix degradation.

Although little is known about the combination of cytokines and granule proteins secreted by neutrophils, their cumulative effect may be responsible for maintaining the proinflammatory environment in ENL. Previous reports showed that the cytokine IL-1β enhances stimulus-induced release of MPO from neutrophils and is associated with tertiary granule exocytosis in both human neutrophil cultures and differentiated HL-60 cells (Dularay et al., 1990; Naegelen et al., 2015b). In our cultures, we did not observe any change regarding IL-1β release mediated by *M. leprae* alone or in the presence of the inhibitors. Naegelen et al., 2015 described by linear fitting, the release of the cytokine IL8 related to CD66b, suggesting the secretion of this cytokine correlated to specific granules, and IL-1β, IL8, and IL6 release were strongly correlated to the marker of tertiary granules. A similar correlation pattern was highlighted in our analysis when comparing the levels of these cytokines with PTX-3 and/or MMP-9. Moreover, IL-6 secretion appears to share pathways with the degranulation of secondary granules, as evidenced by the fact that, inhibition of mitochondrial ROS release and TGF- β treatment weakened its release, but especially p38 MAPK inhibition, which significantly affected this process. This suggests that, although there is currently no consensus on whether the cytokines analyzed are constitutively stored in neutrophil granules and released through degranulation, or instead, are produced exclusively via *de novo* synthesis following activation (particularly IL-1β and IL-6), it is evident that a strong feedback loop exists between neutrophil degranulation and the release of these inflammatory cytokines. In summary, our findings demonstrate molecular mechanisms underlying *M. leprae*-induced degranulation, with p38 MAPK pathway and mitochondrial ROS production involved in this process. Our study also reinforces that the modulatory effect of TGF-β on neutrophil degranulation and LDN generation is done through TGF-β receptor 1 binding, suggesting potential therapeutic strategies to be explored for targeting ENL-related degranulation. Furthermore, additional studies evaluating other signaling pathways will be important to further define the molecular mechanisms underlying neutrophil activation and degranulation in response to *M. leprae*. In addition, the lack of additional phenotypic and morphological characterization of LDNs highlights the need for future studies evaluating whether the effects of the inhibitors are associated with specific LDN subsets, which will be important to better understand neutrophil heterogeneity in response to *M. leprae*. Finally, it remains uncertain whether these findings will be reproduced with other mycobacteria related to leprosy like *M. lepromatosis*, and this knowledge gap should be considered when interpreting the findings.

## Data Availability

The original contributions presented in the study are included in the article/Supplementary Material, further inquiries can be directed to the corresponding author.
