# Supplementary material for: Differential regulation of neutrophil degranulation by p38 MAPK and mitochondrial ROS during Mycobacterium leprae infection
Source: Front Pharmacol. 2026 Aug 12;17:1883257. doi: 10.3389/fphar.2026.1883257 (PMC13506804; doi:10.3389/fphar.2026.1883257)
Supplement: Supplementary file 1 [file Supplementaryfile1.pdf]

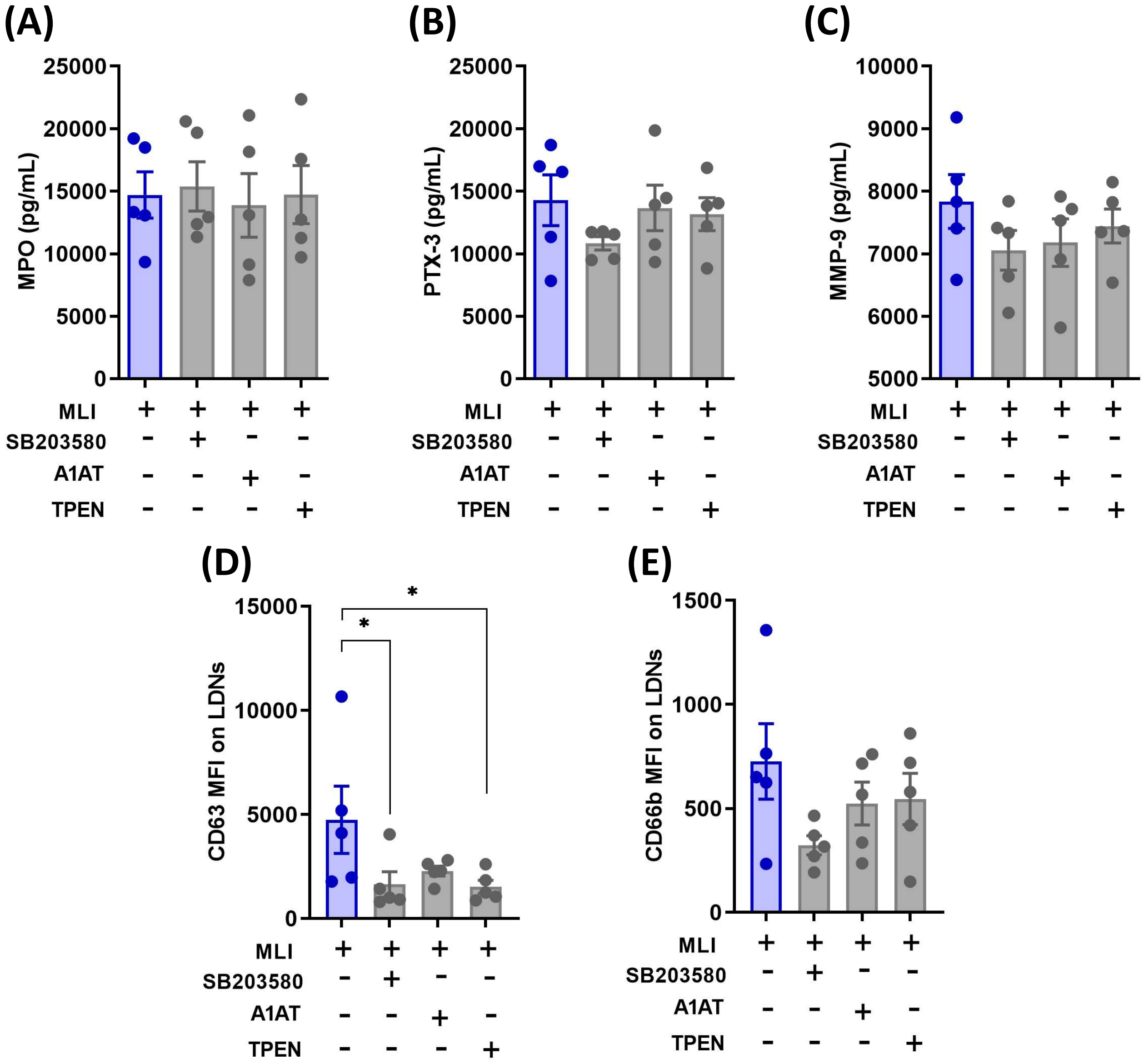

Supplementary Figure S1: Effect of SB203580, A1AT, and TPEN inhibitors on MLI mediated degranulation *in vitro*. ELISA measured granule proteins on plasma culture supernatants. Translocation of degranulation markers to the plasma membrane of LDNs was assessed by flow cytometry. Analysis were done upon pretreatment of whole blood for 30 minutes or not with 10 µM of SB203580, 20 µg/mL of A1AT or 5 µM of TPEN, followed by stimulation with 10 µg/mL of MLI for 24 h. Concentration of (A) MPO, (B) PTX-3 and (C) MMP-9 (pg/mL), in stimulated cultures after pretreatment with SB203580, A1AT or TPEN. Median of fluorescence intensity (MFI) of (D) CD63 and (E) CD66b translocation to LDNs plasma membrane after pretreatment with SB203580, A1AT or TPEN. Data from 3 individual experiments. Each point represents an individual donor and bars represent mean ± SEM per group (n=5). One-way ANOVA followed by Dunn's test. *$p < 0.05$.

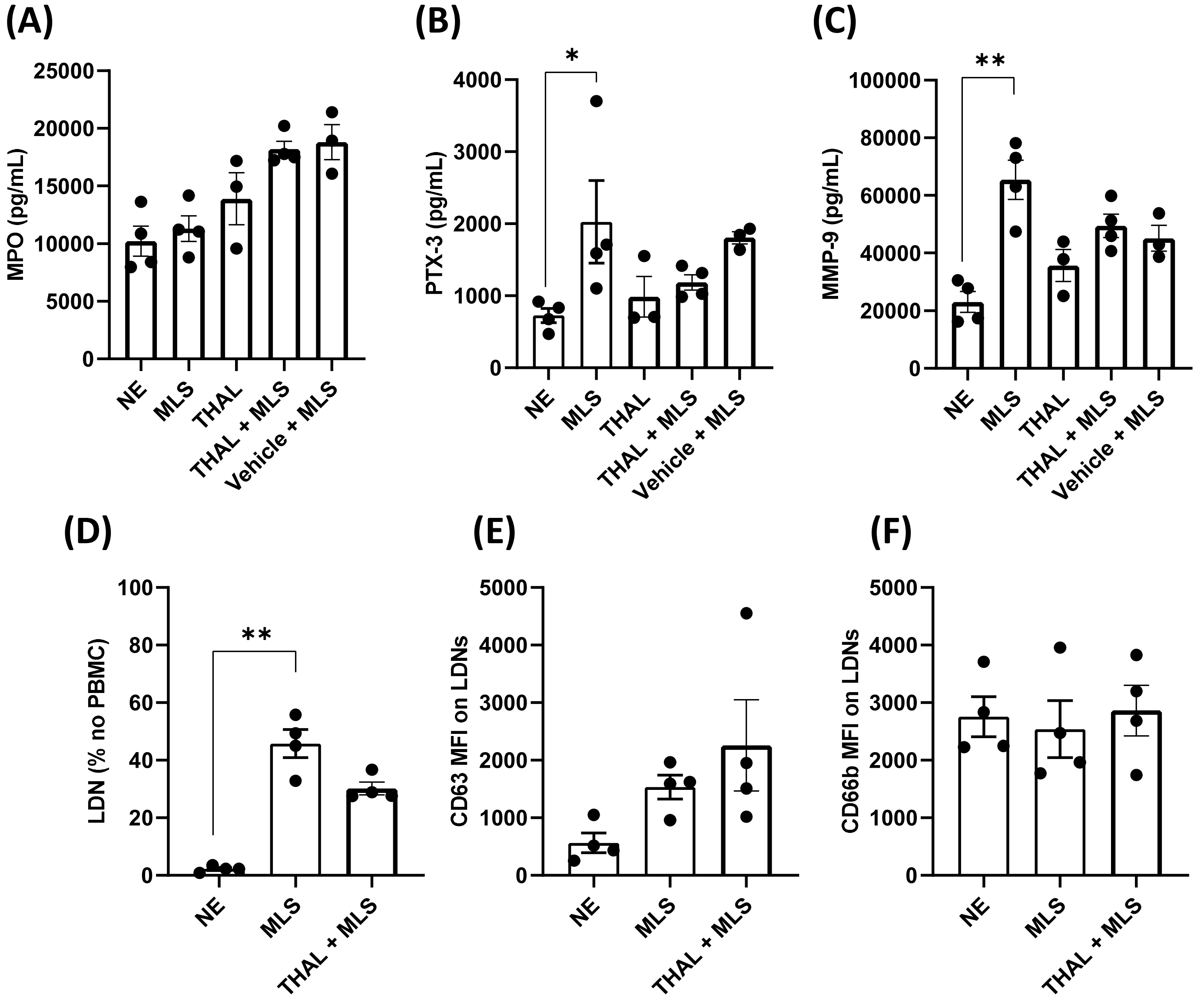

Supplementary Figure S2: Thalidomide effect on MLS mediated degranulation *in vitro.* ELISA measured granule proteins on plasma culture supernatants. Translocation of degranulation markers to the plasma membrane of LDNs was assessed by flow cytometry. Analysis were done upon whole blood stimulation with 10 µg/mL of MLS alone or in combination with 50 µg/mL of thalidomide (THAL) dissolved in DMSO (vehicle) for 24 h. Concentration of (A) MPO, (B) PTX-3 and (C) MMP-9 (pg/ml), (D) Frequency of generated LDNs. Median of fluorescence intensity (MFI) of (E) CD63 and (F) CD66b translocation to LDNs plasma membrane. Data from a single experiment with 4 individual donors. Each point represents an individual donor and bars represent mean ± SEM per group (n=3-4). ANOVA followed by Dunn's test. *p< 0.05, **p< 0.01.
